# *Longissimus dorsi* Muscle’s Chemical Composition, Fatty Acid Pattern, and Oxidative Stability in Korean Hanwoo Finishing Cattle Following Slaughtering and Stunning with or without Brain Disruption and State of Consciousness

**DOI:** 10.3390/foods12050928

**Published:** 2023-02-22

**Authors:** A. B. M. Rubayet Bostami, Hong-Seok Mun, Chul-Ju Yang

**Affiliations:** 1 Animal Nutrition and Feed Science Laboratory, Department of Animal Science and Technology, Sunchon National University, 255 Jungang-ro, Suncheon 57922, Jeollanam-do, Republic of Korea; 2 Department of Animal Science and Nutrition, Bangabandhu Sheikh Mujibur Rahman Agricultural University, Gazipur 1706, Bangladesh; 3 Department of Multimedia Engineering, Sunchon National University, 255 Jungang-ro, Suncheon 57922, Jeollanam-do, Republic of Korea; 4 Interdisciplinary Program in IT-Bio Convergence System (BK 21 Plus), Suncheon National University, 255 Jungang-ro, Suncheon 57922, Jeollanam-do, Republic of Korea

**Keywords:** slaughtering, *Longissimus dorsi* muscle, proximate composition, fatty acid profile, oxidative stability, Korean Hanwoo finishing cattle

## Abstract

Handling during pre- and post-slaughter conditions can affect the quality and safety of meat. An experiment was conducted to compare slaughtering with or without a state of consciousness on *Longissimus dorsi* muscle’s proximate composition, cholesterol content, fatty acid profile, and storage quality (pH, microbiology, and thiobarbituric acid reactive substances (TBARS) value) in Korean Hanwoo finishing cattle (KHFC). Twenty-four KHFC (three replications of four animals per replicate) were slaughtered following two methods: (1) SSUC: slaughtering by applying captive bolt stunning, brain disruption, and neck cutting with the animal in an unconscious state; and (2) SSCS: slaughtering by applying captive bolt stunning, without brain disruption, and neck cutting with the animal in a conscious state. General carcass traits, proximate composition (exempting higher ash content), and cholesterol content of the *Longissimus dorsi* muscle did not differ between slaughter treatments (SSCS vs. SSUS) (*p* > 0.05). The total SFA, UFA, PUFA, and MUFA values did not change for those subjected to different slaughtering types; however, some particular SFA values, namely lauric, myristic, and myristoleic acid, were diminished for the SSCS method as compared with the SSUC method (*p* < 0.05). The *Longissimus dorsi* muscle’s pH value was elevated (*p* < 0.05), the microbial population tended to be diminished (*p* < 0.10), and the TBARS value was suppressed for the SSCS method relative to that of the SSUC method during 2 weeks of storage (*p* < 0.05). Thus, compared with the SSUC method, the SSCS method ensured splendid storage quality with some positive influence on the proximate composition (total ash content) and fatty acid profile (some specific saturated fatty acids) of the *Longissimus dorsi* muscle of KHFC.

## 1. Introduction

Meat is one of the most commonly consumed, first-choice, and protein-rich livestock products in modern consumers’ daily diets. The edible parts obtained from domestic animals, such as bovine, ovine, porcine, poultry, and wild game, are considered to be “Meat” according to the European Commission (EC) (Regulation EC 835/2004). The literature has reported that meat can be the whole or part of the carcass of any buffalo, cattle, goat, sheep, deer, camel, hare, rabbit, or poultry after slaughtering, other than in the wild state, where eggs and fetuses are not included according to FSANZ [1]. There are, in general, two types of meat, red and white meat: the meat obtained from cattle, goat, sheep (e.g., beef, veal, mutton, lamb, and goat meat), and pig (e.g., pork, ham, and bacon) are considered to be red meat, and poultry meat or muscle obtained from fish are considered to be white meat. 

Although there are controversies regarding the association of red meat with cardiovascular disease, there are many positive impacts of red meat consumption in the daily diet. Red meat not only contains major nutrients such as protein or amino acids, but also a range of fats, namely, essential omega-3 polyunsaturated fats, and low fat and moderate cholesterol (lean red meat), as well as many essential minerals, vitamins, antioxidants, and bioactive compounds [2,3]. All essential amino acids are present in red meat, such as lysine, methionine, tryptophan, threonine, leucine, valine, and isoleucine, and there are no limiting amino acids [2]. Additionally, niacin, vitamin B6, vitamin B12, riboflavin, pantothenic acid, potassium, sodium, calcium, zinc, phosphorus, iron, and selenium are present [2]. Beef obtained from cattle contains predominantly conjugated linoleic acid (CLA) isoform (cis-9, trans-11 CLA, or rumenic acid), which shows anticarcinogenic effects and causes no alteration of body composition, whereas trans-10 and cis-12 CLA inhibit lipogenesis [4]. Low levels of conjugated linoleic acid can increase the risk of cancer; however, a sufficient amount of CLA can prevent cancer [4].

The digestibility of red meat is higher than that of plant protein (94% for red meat, 86% for whole wheat, and 78% for beans) [5]. Muscles from beef, pork, chicken, and marine animals are sources of bioactive compounds, including a myriad of peptides, namely antioxidant, opioid, antihypertensive, antithrombotic, and other bioactive compounds [3]. Meat consumption should include the concept of functional foods because meat contains high-quality proteins and bioactive peptide compounds. The proportion of amino acids can differ among different animal species, within the same species, and across different locations of the animal body. Susanto et al. [6] stated in their review that more than 170 peptides could be released from the main structure of myofibrils (actin, myosin) and sarcoplasmic muscle proteins of different animal species (e.g., cattle, pigs, or poultry), where the synthesis, extraction, and identification of bioactive peptides in meat, as well as their potential use as functional foods, has been gaining interest. 

Meat is obtained from the industry through a slaughtering process involving pre- and post-slaughter care and handling. Management, pre- and post-slaughter activities, and handling can affect the qualitative and quantitative characteristics, safety concerns, consumer choice, demand, and ritual matters of the meat [7,8]. The cattle industry of the Republic of Korea has aimed to ameliorate meat production ability and to boost the total quantity of cattle to attain the appeal of the flourishing beef market [9]. The per capita consumption of meat in the Republic of Korea has rapidly grown from 46 kg in 2012 to 68 kg in 2019 (Statista, 2022), where beef consumption increased to 12 kg from 3 kg (KAPE, 2011, Korea Institute for Animal Products Quality Evaluation). The Republic of Korea is momentous for its rapid economic augmentation from an underdeveloped empire to a developed money-making state in a trifling generation purported as the Miracle on the Han River [10], permitting the Republic of Korea to become an OECD member country. In addition to this, the Republic of Korea has been placed in the group of the Next Eleven countries having the ability to take leading action in the national and global economy by the G-20 (Goldman Sachs, 2021). Owing to such remarkable economic development, quantity, as well as the demand for safe and high-quality meat, has increased all over the Republic of Korea. In contrast, due to globalization, the number of people from different religious and ethnic groups is also increasing in the Republic of Korea which creates a large open market opportunity for different consumable food items as well as meat and other animal-derived products. 

Food choice normally reflects aspects of economic status, lifestyle, culture, ethnopolitics, religion, diet category, and health concerns. Jews prefer foods pertaining to Judaism’s rules and regulations, and Christianity, Buddhism, and Hinduism also accept and reject foods based on their religious taboos. Muslims select foods according to the Sharia Law of Islam, where the judgment to choose one food over another depends on halal or ritual issues. There are different types of slaughtering systems such as halal, kosher, sikh, and some other ritual methods; however, mostly halal or kosher methods are applicable to the global meat market, and are practiced by Muslims and Jews, respectively. Nevertheless, practices being performed in the meat industry can affect the quality and safety aspects of meat. It is ascribed that different factors, namely the management of pre- and post-slaughter practices, can influence the composition and quality of meat obtained from the animal. The genetic make-up, types of diet, age, sex, stage of production, management, and pre-slaughter handling and care can affect the carcass quality, meat composition, and fatty acid profile in animals [11,12]. Pre-slaughter activities (loading and bullying of animal by others) and season or breed differences can influence the qualitative parameters of meat (pH, color, thawing loss, cooking loss, WBSF values, or the toughness of meat) and the fatty acid profile [13,14].

According to our review of the literature, there is scarce research on slaughtering followed by captive bolt stunning, brain disruption, and state of consciousness (conscious vs. unconscious) in cattle. The full neck cutting following captive bolt stunning in the unconscious state (fully dead condition) in the hanging condition is the common practice in the Korean cattle slaughterhouse, and in some other countries, where the slaughtering process is considered as non-halal (conscious state of animal or bird is essential to fulfil the halal meat criteria). To attain the national and international consumers’ demand, it is required to introduce and adopt some convenient slaughtering method with little variation with the existing slaughter practice, so that local communities’ manners are not hampered and dishonored. Notably, the global ritual or halal meat business can be captured and the regional demand for ritual meat can be fulfilled through the adaptation of the operating slaughtering practice. Therefore, the present experiment was conducted to compare slaughtering and captive bolt stunning with or without brain disruption and conscious state of animal on the *Longissimus dorsi* muscle’s proximate composition, cholesterol content, fatty acid pattern, and storage quality (pH, microbiology, and thiobarbituric acid reactive substances (TBARS) value).

## 2. Materials and Methods

### 2.1. Animal Transport, Lairage, Stunning, Slaughtering, and Processing

A total of twenty-four Korean Hanwoo finishing cattle (KHFC) castrated mature males were selected based on similar age (30.0 ± 1.0 months) and body weight (735.36 ± 28.93 kg) range groups for two types of slaughtering, where for each category twelve cattle were slaughtered and triplicate samples were taken from each carcass. The KHFC were reared in Naju city, the Republic of Korea commercial ranch, which provided a commercially available corn-based concentrate diet, rice straw, and hay to meet the nutritional requirements of growing finishing cattle. The crude protein content of the concentrate diet that was provided to the finishing cattle was 11.6%, while the approximate body weight of the cattle was 450 ± 12.0. The experimental slaughtering was conducted in the abattoir of Naju city, the Republic of Korea. The animals were slaughtered with different methods to compare the quality of the *Longissimus dorsi* muscles obtained from KHFC. The experiment was conducted to compare the slaughtering with state of consciousness on the *Longissimus dorsi* muscle’s chemical composition, cholesterol content, fatty acid profile, and storage quality (pH, microbiology, and TBARS value) in Korean Hanwoo Finishing Cattle (KHFC). The KHFC animals were slaughtered following two methods: (1) SSUC: slaughtering by applying captive bolt stunning, brain and spinal cord disruption, and neck cutting with unconscious state of animal and (2) SSCS: slaughtering by applying captive bolt stunning, without brain and spinal cord disruption and neck cutting with conscious state of animal. All guidelines for the care and use of animals in research set by the Korean Ministry for Food, Agriculture, Forestry and Fisheries (2008) were followed for the present study. The care and management of animals and experimental works was followed according to the Animal Care and Management Committee of Sunchon National University, the Republic of Korea. Ethical approval for animal and animal-derived product quality testing was obtained (SCNU IACUC-2017.06), where all rules and regulations were followed according to the guidelines of the Ministry for Food, Agriculture, Forestry and Fisheries, the Republic of Korea, and Sunchon National University, the Republic of Korea. 

The KHFC which were subjected to being slaughtered were transported following animal welfare guidelines. After arrival of the KHFC at the abattoir during the early morning, they were rested in a lairage pen to reduce stress and settle them for about 1 h. Prior to moving the animals in the abattoir, all animals were off feed for 24 h with ad libitum access to water. The KHFC were randomly moved to the pen for stunning by captive bolt following body weight checking of each. It is a well-known fact that stunning is the process of rendering animals immobile or unconscious, without killing the animal, prior to them being slaughtered for food. For both of the treatments, animals were randomly selected and stunned by captive bolt (manufactured by Accles and Shelvoke, Sutton Coldfield, West Midlands, UK). In the case of the SSUC method, the animals were stunned by captive bolt; brain and spinal cord disruption was carried out by applying a metal rod to the brain of the cattle to confirm a dead or unconscious state, and a sharp knife was thrust into the chest for bleeding for 60 s. Finally, the whole neck was cut and separated from the body without applying any specific rules of slaughtering.

In the SSCS method, the neck was cut following captive bolt stunning, no brain and spinal cord disruption was applied, and the consciousness state of the animals was assured by skilled technical personnel. The trachea, brachial veins, jugular vein, and esophagus were cut within a short stipulated time by a skilled slaughtering person with the help of a sharp knife, following a required stroke. No brain and spinal cord disruption was applied in the head region and the sharp knife was not thrust into the chest for bleeding. After cutting the neck without separating it from the body, it was bled out for 60 s. After permitting the bleeding, the whole head was separated from the body and went through further processing such as dehiding, evisceration, and dressing. During captive bolt stunning, care was taken to place the captive bolt pistol firmly against the skull and the firearm was held 5 to 20 cm away from the skull following Grandin [15] guidelines. The pre- and post-slaughter care and inspection, stunning, slaughtering, and bleeding of animals were inspected and practiced by a technical person. In both cases, slaughtered animals were hoisted by shackle and then hide and legs was separated in the abattoir. Following evisceration and dressing, the carcass was divided equally into two parts. The carcass was left in the chilling room for 24 h at around 2 to 4 °C following proper washing and cleaning with care taken. Following proper chilling, the *Longissimus dorsi* sample was collected for further analysis. 

The SSCS cutting of the neck of the animal was followed based on the Islamic halal slaughtering principle where the guidance is in accordance with the Quran (Malaysian Halal Standard, 2009, MS:2009). For slaughtering an animal, it should usually pass through a specific regulation of living state to a dead state. Meat that will be lawful (halal) according to the Sharia Law of Islam must be obtained from a lawful listed animal species, must be ritually slaughtered by a good practicing person, and blood should be drained properly, i.e., according to the Sharia Law, or the sole code should be recognized by the group as legitimate. The slaughter was therefore performed by a good practicing Muslim person by reciting the name of Almighty Allah (Bismillah Allahu Akbar) and blood was drained out of the carcass in the proper way; it was confirmed by visual observation that the animal was alive prior to slaughter by a technical person. Proper inspection of all animals was carried out to check the health status or any deformities. During handling and transportation, welfare aspects were considered. Before the slaughtering of the animals, enough rest was provided, stress-free conditions were ensured, and they were provided with water overnight. After proper slaughtering of individuals, evisceration was properly carried out and the hygienic conditions of the whole carcass were maintained. The overall physical and health condition was inspected by a veterinarian. The live weight and carcass weight of the animals were taken before and after slaughter. Following stunning and slaughtering, skin removal and evisceration were practiced and after washing they were immediately transferred to the chilling room, maintaining the temperature of 2 °C. 

### 2.2. Carcass Handling and Sample Preparation 

After 24 h, the left side of the carcass was ribbed between the twelfth and thirteenth rib. The quality grade (MQG) and yield grade (MYG) of the KHFC carcass was determined by experienced officials. The Korean carcass grading system has been applied since 1992 to the meat industry, having the quality and yield grades: quality grades indicate five values including 1++, 1+, 1, 2, and 3; and the yield grade indicates A, B, or C, the three possible values. The MQG is dictated by the marbling score, the maturity score of the *Longissimus dorsi* muscle at the 12th to 13th rib interface, fat and meat color, and firmness and texture of meat [16], where the MYG is dictated by the carcass weight, loin eye area, subcutaneous fat thickness, and maturity. Grade 1++ indicates the highest quality grade, while Grade 3 indicates the lowest quality grade [16]. The quality grade (MQG) and yield grade (MYG) of the KHFC carcass were applied by following the guidelines of NLCF [16] (National Livestock Cooperatives Federation; Republic of Korea, 1998) and elaborate elucidation of Bostami et al. [17], Korean Beef Carcass Grading Standards (KAPE, 2012, Korea Institute for Animal Products Quality Evaluation), and the Korean Carcass Grading Procedure [16].

Following grading, the carcasses were taken to the cutting room and then the *Longissimus dorsi* samples were collected from the 12th to 13th thoracic vertebrae from both sides of the carcasses. Three replicates of *Longissimus dorsi* muscles were taken from each individual of the KHFC carcass. After collection of the *Longissimus dorsi* sample, trimming for subcutaneous fat, peripheral muscles, and epimysium was performed carefully. All *Longissimus dorsi* samples were properly prepared and stored carefully for further specific analysis. Then, the *Longissimus dorsi* samples were divided for proximate composition analysis, fatty acid pattern analysis, cholesterol content analysis, physico-chemical analysis, and storage quality analysis (pH, microbiology, and TBARS value). *Longissimus dorsi* samples were tested and analyzed in the Laboratory of Meat Science, Department of Animal Science and Technology, Sunchon National University, the Republic of Korea.

### 2.3. Proximate Composition Analysis of Longissimus dorsi Muscle

To determine the composition of the experimental KHFC *Longissimus dorsi* muscle, the samples were then properly excised and grinded with the help of a grinder (Ultra Turrax, IKA works Inc., Staufen im Breisgau, Germany). For the determination of moisture content, crude fat content, crude protein content, and crude ash content, the procedures and guidelines of AOAC [18] were applied. 

### 2.4. Cholesterol Content Determination of Longissimus dorsi Muscle

The cholesterol content of the *Longissimus dorsi* muscle of KHFC was determined. The cholesterol determination was derived from fat, which was separated via the extraction of 5 g of minced meat (mixed with reference material; 0.5 mL of 5α-cholesterol) with a chloroform and methanol mixture (2:1 vol:vol) following the procedure and guidelines of Folch et al. [19]. Cholesterol was separated from fat after saponification with KOH and extraction with ethyl ether by the modified method described by King et al. [20] and Ahmed et al. [21]. 

### 2.5. Fatty Acid Determination of Longissimus dorsi Muscle

The fatty acid composition of the *Longissimus dorsi* muscle of KHFC was determined using a direct method for fatty acid methyl ester (FAME) synthesis using a slight modification of the method described by O’Fallon et al. [22] and Ahmed et al. [21]. For evaluating nutritional value and healthiness of the fatty acid profile, the sums and ratios were determined. The sum of the saturated fatty acids (SFAs), mono-unsaturated fatty acids (MUFA), polyunsaturated fatty acids (PUFA), n-3 fatty acid (n-3), and n-6 fatty acids (n-6) was determined, where the ratios of UFA: SFA, PUFA: SFA, n-6: n-3, and hypocholesterolaemic: hypercholesterolaemic fatty acid were determined. The H/H ratio was determined according to Santos-Silva and Santos-Silva [23] and Ahmed et al. [21]. Since some of the data were not significant, not all of the data were shown in the results, especially n-6: n-3 and hypocholesterolaemic: hypercholesterolaemic fatty acid.

### 2.6. Measurement of Longissimus dorsi Muscle’s pH

The pH of the *Longissimus dorsi* muscle was measured using a digital pH meter (Docu-pH+ meter, Sartorius, Bohemia, NY, USA). The *Longissimus dorsi* muscle sample (4 g) was measured in a Falcon tube, after which deionized water (36 mL) was added, and then it was homogenized properly (30 s, 13,000 rpm, 25 °C) for mixing and filtered. Following homogenization and filtering, the pH value of the slurry was determined and recorded.

### 2.7. Longissimus dorsi Muscle’s Microbiological Analysis

To evaluate the meat microbial growth, *Longissimus dorsi* muscle samples were taken with three replicates from the carcass of the KHFC. A 25 g *Longissimus dorsi* sample was homogenized by adding 225 mL of 0.85% (*w*/*v*) NaCl solution. Thus, a 1:10 dilution of the sample was obtained; after which, 10-fold serial dilutions (10^−2^ to 10^−13^) were assembled by utilizing 0.85% NaCl solution. Dilutions were adopted following the recommended guidelines of the International Organization for Standardization (ISO, 1995). Then, 20 µL (instead of 100 µL) from serial dilutions was transferred with the help of a sterilized micro-pipette, and was spread onto Tryptic soy agar plates (Becton, Dickinson and Company, Sparks, MD 21,152, USA) with the help of a sterilized triangle-spreader for microbial enumeration. Duplicate plates of each dilution were incubated at 37 °C for 48 h, and the following incubation colonies were counted promptly. The calculations were made using a Microsoft Excel file after data entry. After counting the microbial colony, the microbial number was calculated as follows: number of colony × 10^n^ × (100/20) = multiplied value = log (multiplied value) (n: dilution value, e.g., 1 to n), where the calculated value of the microbial colony count was expressed as log_10_CFU/g.

### 2.8. Longissimus dorsi Muscle’s Oxidative Stability Analysis

To determine the oxidative stability of the *Longissimus dorsi* muscle, samples were preserved in a refrigerator at 4 °C for 1 day to 15 days, and after which the thiobarbituric acid reactive substance (TBARS) values were determined. As a brief description, 4 g of *Longissimus dorsi* muscle sample was properly homogenized with the help of a homogenizer (Ultra-Turrax T-25 Basic, IKA Werke, GMBH & CO. KG, Staufen, Germany), where the full speed was applied for 1.5 min with 10 mL of a solution containing 20% trichloroacetic acid (TCA) in 2 M phosphoric acid and 10 mL distilled water. Then, Hyundai Micro No. 60 (Hyundai Micro Co., Ltd., Seoul, the Republic of Korea) filter paper was used to filter the mixture. Then, a proportionate volume of the filtrate (2 mL) and 2-thiobarbituric acid (98% 4, 6 dihydroxy-2-mercaptopyrimidine, 0.005 M in DW) were heated properly in a shaking water bath maintaining 80 °C for 30 min. Following cooling, the absorbance was measured using a spectrophotometer, where it was set at 530 nm with a VIS-Spectrophotometer (Libra S22, Biochrom Ltd., Cambridge, UK). After measuring the absorbance or optical density value (OD value) of the standard and each sample of the treatments, the value of TBARS was calculated by the following formula: the amount of TBARS was calculated and expressed as a micromole malondialdehyde (µmol MDA) per 100 g of meat.

(1) $$\mathrm{TBARS}\;\mathrm{value}=\{\frac{\mathrm{OD}\;\mathrm{value}\;\mathrm{of}\;\mathrm{sample}-\mathrm{OD}\;\mathrm{value}\;\mathrm{of}\;\mathrm{standard}}{\mathrm{Weight}\;\mathrm{of}\;\mathrm{sample}}\}\times 3\times 100\;$$

### 2.9. Statistical Analyses

All experimental data were recorded, arranged, and curated for further statistical analysis, where analysis of variance was followed using the general linear model (GLM) function of the statistical analysis system (SAS, 2012, SAS Institute, Cary, NC, USA). The statistical significance was considered at the level of *p* < 0.05, where the level of *p* < 0.10 was contemplated as the statistical tendency. A triplicate replication was followed for all measurements and analyses where a duplicate sample for each dilution was analyzed for microbial analysis. The statistical model which was followed for the current study was to test the impact of slaughtering type on the general carcass parameters, *Longissimus dorsi* muscle proximate composition analysis, fatty acid pattern analysis, cholesterol content analysis, and storage quality analysis (pH, microbial load, and TBARS) in the case of KHFC.

*Y_ij_* = *µ* + *α_i_* + *e_ĳ_* (2)

where *Y_ij_* = the response variable, *µ* = the general mean, *α_i_* = the effect of slaughtering treatments, and *e_ĳ_* = the random error. Student’s *t* test with a probability level of *p* ≤ 0.05 was used for the comparison of mean values. The correlation, RSQ, and intercept were calculated using MS Excel software 2016.

## 3. Results 

### 3.1. General Carcass Traits

Table 1 shows the general carcass characteristics of KHFC that were slaughtered following two types of slaughtering with or without a state of consciousness of the animal. The results elucidated that general carcass traits were in a similar range between the slaughter groups of KHFC. 

### 3.2. Longissimus dorsi Muscle’s Proximate Composition, Cholesterol Content, and Fatty Acid Profile

**Figure 1:**
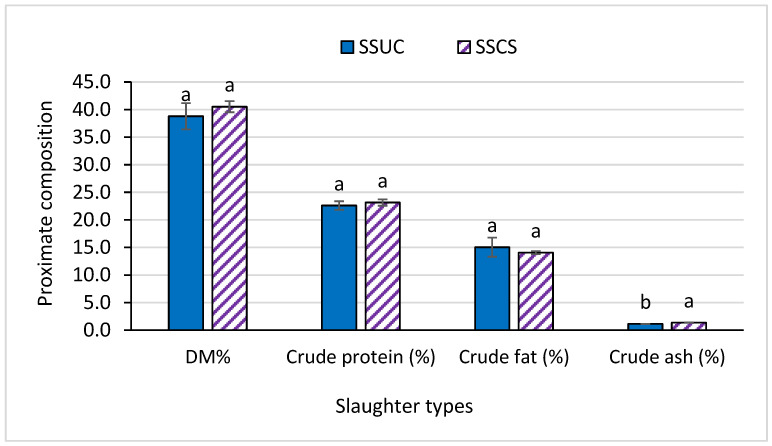
Effect of slaughtering with or without state of consciousness on *Longissimus dorsi* muscle’s proximate composition in Korean Hanwoo Finishing Cattle. ^a, b^ Means in the same bar with different superscript letters are significantly different at *p* < 0.05. Korean Hanwoo Finishing Cattle (KHFC) were slaughtered following two methods: (1) SSUC: slaughtering by applying captive bolt stunning, brain and spinal cord disruption, and neck cutting with unconscious state of animal and (2) SSCS: slaughtering by applying captive bolt stunning, without brain and spinal cord disruption and neck cutting with conscious state of animal.

**Figure 2:**
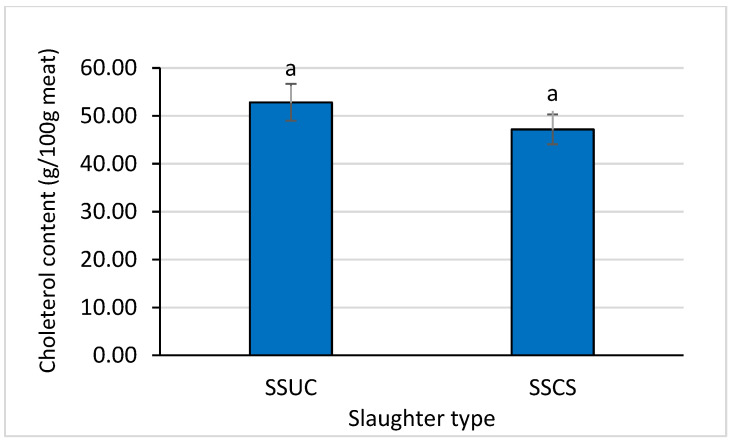
Effect of slaughtering with or without state of consciousness on *Longissimus dorsi* muscle’s cholesterol content in Korean Hanwoo Finishing Cattle. Bar diagram with similar superscript letters are not significantly different at *p* < 0.05.

The *Longissimus dorsi* muscle’s proximate composition data revealed no differences in the case of dry matter, crude protein, and crude fat content, whereas the ash content differed in the SSCS group relative to the SSUC group (*p* < 0.05) (Figure 1). The cholesterol content (Figure 2) of the *Longissimus dorsi* muscle was not affected by the slaughtering types (values of cholesterol were 52.83 vs. 47.17, *p* > 0.05). As shown in Table 2, among fatty acids, lauric acid (C12:0), myristic acid (C14:0), and myristoleic acid (C14:1n5) were diminished in the SSCS group in comparison to the SSUC group (*p* < 0.05). However, the total SFA, UFA, PUFA, and MUFA and the ratios of SFA/UFA, SFA/PUFA, and SFA/MUFA did not differ significantly between the groups (SSCS vs. SSUC) (*p* > 0.05).

### 3.3. Longissimus dorsi Muscle’s Storage Quality (Microbial Loads, pH, and Oxidative Stability)

**Figure 3:**
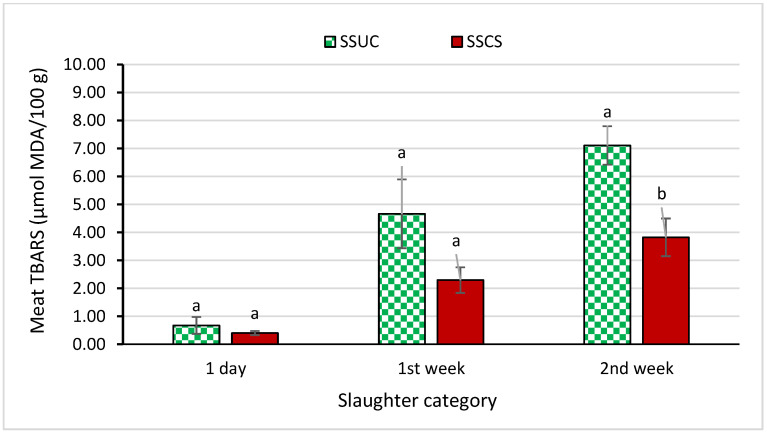
Effect of slaughtering with or without state of consciousness on *Longissimus dorsi* muscle’s oxidative stability (TBARS value) in Korean Hanwoo Finishing Cattle during storage at 4 °C. ^a, b^ Means in the same bar with different superscript letters are significantly different at *p* < 0.05.

The storage quality data of the *Longissimus dorsi* muscle suggested that slaughtering differences affected the *Longissimus dorsi* muscle’s pH, microbiology, and oxidative stability (TBARS value) to some extent. In Table 3, it can be observed that the storage *Longissimus dorsi* muscle’s pH value was upgraded in the SSCS group relative to the SSUC group during the 2nd week of storage (*p* < 0.05). A tendency of a lower microbial population was observed in the SSCS group as compared to the SSUC group during the 2nd week of storage period (*p* < 0.10) (Table 4). As shown in Figure 3, the *Longissimus dorsi* muscle’s TBARS value during the 2nd week of storage was diminished in the SSCS group compared to the SSUC group (*p* < 0.05).

### 3.4. Correlation, RSQ, and Intercept among pH, Microbial Loads, and TBARS Value

The correlations among pH, microbial loads, and the TBARS values are shown in Table 5. The correlation value between the pH and microbial loads of the *Longissimus dorsi* muscle reveals that during the 1st week there was a positive correlation, whereas on Day 1 and the 2nd week of storage there was both a positive and negative correlation between slaughtering following stunning with or without brain and spinal cord disruption and conscious state of animal. The pH and TBARS correlation value depicted that on Day 1 there was a negative correlation, whereas during the 1st and 2nd weeks of storage there was a positive correlation. The correlation between microbiology and TBARS indicated that there was a negative and positive correlation on Day 1, a negative correlation during the 1st week of storage, and a positive correlation during the 2nd week of storage period between slaughtering following stunning with or without brain and spinal cord disruption and conscious state of animal.

The RSQ values among pH, microbial loads, and the TBARS values are shown in Table 6. RSQ usually indicates the amount of discrepancy in the dependent variable (Y) that is elucidated by the divergence in the independent variable (X). One should not be persuaded to keep adding variables to uplift the RSQ value. It is indicated that they may not always be significant and will not certainly aid in a specific prediction. In a regression model, R-squared is a statistical means of fit that actually demonstrates that the degree of deviation of a dependent variable which is elucidated by the independent variable(s). The RSQ value in response to slaughtering following captive bolt stunning with or without brain and spinal cord disruption and state of consciousness showed both higher and lower values, indicating a good fit of the data set among the pH, microbiology, and TBARS value of the *Longissimus dorsi* muscle.

The intercept among pH, microbial loads, and the TBARS values of the *Longissimus dorsi* muscle are shown in Table 7. In the current study, the intercept was calculated to find the statistical relationships and data set standards among the pH, microbiology, and TBARS values of the *Longissimus dorsi* muscle. The intercept value in response to slaughtering following captive bolt stunning with or without brain and spinal cord disruption and the state of consciousness showed both a higher and lower value; however, there was no negative value. Therefore, we assumed that the data set was good when comparing the pH, microbiology, and TBARS values of the *Longissimus dorsi* muscle.

## 4. Discussion

### 4.1. General Carcass Traits

Meat is commonly consumed as fresh or processed products. Meat is obtained after the slaughtering process from an industry in which a chain of processes is maintained. The mechanism of transformation of muscle to meat might vary from region to region based on socio-political and other issues such as national wealth, security, poverty, civilization, income inequality, religious matters, social integration, political conflicts, and ritual factors [24,25]. Different steps and factors are associated with meat to achieve the consumer’s satisfaction at the table. Stunning, pithing, brain and spinal cord disruption, breast sticking, neck cutting, and bleeding are the steps during the slaughtering process to make the meat available from common livestock or poultry species. The main purpose of stunning is making the animal unconscious prior to slaughtering and pithing influences the animal to die through physical interference of the brain and the rostral part or the anterior part of the spinal cord, and prevents uncoordinated movements [26]. Poultry, rabbits, pigs, and sheep are commonly stunned by applying the electrical method; cattle are mostly stunned using a captive bolt stunner [27,28]. 

People’s perspectives in relation to the killing of animals and meat consumption are shaped by spirituality and ideology. Consideration of the animal species, sources of meat, methodology applied, and the death state of the animals add another dimension to the quality and safety of meat, which are not possible to evaluate by the explanation of science or measure using any instruments; therefore, down-grading on such types of attributes can have a remarkable impact on the value of meat [29,30]. Stunning and slaughter types can affect the blood loss, meat composition, quality and safety concerns of carcasses as well as meat. It was stated [31] that there was no difference in blood loss in captive bolt stunning with non-halal slaughtering and Muslim halal slaughtering with stunning in cattle. A subsequent study [17] reported that stunning and slaughtering without pithing conferred no negative concussion on the qualitative aspects of meat, and rather led to a higher concentration of loin eye total ash content. In this study, slaughtering after captive bolt stunning with or without brain and spinal cord disruption and the conscious state of the animal were compared. The general carcass traits were in a similar range; thus, they could not substantially affect the other studied factors. In this study, general carcass traits did not differ in response to two-neck cutting after stunning, with or without brain and spinal cord disruption and striking with the animal in a conscious or unconscious state. 

### 4.2. Longissimus dorsi Muscle’s Proximate Composition, Cholesterol Content, and Fatty Acid Profile 

A negative association between intramuscular fat and moisture content was reported in previous studies in the cases of bovine muscle [32,33]. However, in the current study, no relationship was noticed between the KHFC *Longissimus dorsi* muscle’s moisture content and the fat content. Meat is an important source of vitamins and minerals [34]. The results indicated that the slaughtering method did not affect the moisture, crude protein, or crude fat content; however, it did affect the crude ash content, with higher content being observed in the SSCS group than that of the SSUC group, which might be beneficial to the health aspects. The breed, sex, age, production system, and dietary supplementation can affect the ash content of meat [35,36]. Ash content can be elevated whereas moisture content can be decreased with the advancement of slaughter age, where with a downtrend in moisture, the fat content can be upgraded [36,37].

Meat composition and quality can be influenced by the aggregated impact of chronic and continued categories of stressors related to environment or conditions [38]. Among stressors, pre-slaughter handling, transport, and stunning can affect the meat quality, as reported in previous studies [38,39]. The ionic change through stress factors could be a possible link for the variation in the ash or mineral content of muscle between the groups due to variation in slaughtering and conscious state [40]. However, the present study suggested that slaughtering with stunning and with or without physical brain disruption, breast striking, and state of consciousness can affect the composition, and especially the ash content, of meat. The ash content of meat depends on the diet, genotype, age and sex of an animal, castration, geographic conditions, and ritual slaughtering and processing [41,42,43]. In the case of ritual slaughter, the most effective bleeding of carcasses is practiced; that is why in terms of quality and hygiene it is usually the best suited for human consumption. It is stated [44] that the meat obtained from ritual slaughter generally is more durable and does not spoil quickly—it contains less blood and more nutrients. Differences in histology, functions performed in the body, muscle type, and intensity of work during life of the animal can influence the concentration of individual nutrients [45,46]. However, the actual reason for differing ash content in the current study is not clear; the mechanism could be discovered through further detailed study. The cholesterol content was not affected due to differences in the slaughtering system, whereas the fatty acid profile was influenced to some extent by the slaughtering with stunning and with or without physical brain disruption, breast striking, and state of consciousness. Age at slaughter, subspecies, and muscle have no impact on cholesterol content; however, they can affect the fatty acid profiles of ostrich or cattle [47,48,49]. 

In this study, *Longissimus dorsi* muscle was compared following differences in slaughtering steps. The cholesterol content of meat and the intramuscular fat content possess a positive correlationship [50,51]. The lower content of SFA (C12:0, C24:0, and C14:1n5) in the SSCS group compared to that of the SSUC group indicates the better commercial value of KHFC meat with a positive impact, since it has been preached to the world over many years that the diminishing of SFA and the elevation in PUFA in the diet of humans can contribute to the health of humans through the maintenance of blood cholesterol and other factors. Studies have shown that fatness can affect the fatty acid composition [12,52]. Notwithstanding this, there were no significant differences; the backfat thickness and marbling score showed higher values in the SSCS group than the SSUC group, which might be linked with the fatty acid composition. However, the ratio of PUFA to SFA was found to be low in both groups of the present study, which supports the statement of a low ratio in the meat of ruminants [52]. Genetic and nutritional factors, age, stage of fattening, anatomical position, and pre-slaughter handling can affect the fatty acid content of animals [11,12]. It is stated that pre-slaughter activities, loading, and stunning, etc., can influence the meat quality and fatty acid profiles [13,14]; however, to what extent slaughtering differences affect the fatty acid content in KHFC in this study is not clear, conferring further detailed study to explore the actual mechanism. 

### 4.3. Longissimus dorsi Muscle’s Storage Quality (Microbial Loads, pH, and Oxidative Stability)

The quality of the storage of meat depends on several factors, namely meat pH, microbial growth, types of bacteria, oxidative stability, enzymatic activity, and others. The growth of different bacteria can deteriorate the quality of meat. The type of microbial species, the characteristics of meat, the composition, the processing methods, and the storage environment can affect the spoilage of meat, which can also affect the meat pH as well [53,54]. Although great diversity in bacteria was reported in spoiled meat in previous studies [55,56], there exists a close association between predominant bacteria (*B. thermophacta*, *Enterobacriaceae*, *Carnobacterium* spp., *Lactobacillus* spp., *Pseudomonas* spp., *Leuconostoc* spp., and Sh. *putrefaciens*) and the spoilage condition of refrigerated beef [53]. Additionally, it was postulated that the genus or species of lactic acid bacteria is important in meat spoilage based on the composition and processing [55]. Different research has focused on the dimension of microbes on meat quality. The microbial count may vary based on their types and species, where around 10% of the initial bacteria are able to multiply during refrigeration of several days up to several months [57,58,59]. 

Lactic acid bacteria cause discoloration, sour or off flavor, and gas and slime production, and diminish the pH value, lactic acid, acetic acid, and formic acid generation during the refrigeration stage, which results in the spoilage of meat [53,55,60]. The association of amount of retention of blood in the muscle of the carcass with the microbial count was stated in the case of broiler [61] and the condition was explained in the case of rabbit research [62], because the growth and development of spoilage microorganisms is favored by the retention of blood [63]. During the fact-finding of slaughtering methods of a different category, such as the Islamic slaughtering, conventional neck cutting, decapitation, and bled or un-bled method, it was reported by Addeen et al. [64] that there was a diminished total viable microbial count in the Islamic slaughter group in the case of chicken. In the current investigation, although it was not significantly different, the estimated blood weight was higher in the case of SSCS than that of SSUC; however, there might be some link with blood output with the microbial load in the muscle due to reduced blood retention in the muscle following slaughtering with stunning with or without brain disruption, breast striking, and state of consciousness.

The normal pH of muscle is between 5.4 and 5.8 in the post-mortem period, where a higher pH could be associated with the stress to the animal, and it causes the deterioration of lower quality meat [53]. The generation of chemical substances from the microorganism might depend on their type and species, and the storage environment, which can influence the pH as well as the spoilage of meat while in storage for a long period [53,60]. During the storage condition, volatile bases can be generated by the action of microbes which can consequently influence the elevation in the muscle pH value [65], while the growth of lactic acid bacteria can result in the suppression of the pH value and the spoilage of meat during the storage period [53,55,60]. Therefore, the variation in the pH value between groups in the current study during storage might be attributable to the type and species of microorganism in the storage meat. A negative correlation between bacterial count and pH value was reported due to metabolism caused by lactic acid bacteria, and generation of organic acids from the available glucose [66,67,68]. Juncher et al. [69] and Calvo et al. [70] reported that a lower pH of muscles was associated with a higher TBARS value, because a reduction in pH can accelerate the lipid oxidation process due to the elevation in hemoglobin oxidation [71]. There is a close relationship between microbial growth and meat oxidation or spoilage in terms of the counts during the initial stage and their subsequent growth phase [53]. 

In the case of post-mortem, meat lipid oxidation occurred which can be the main impediment to cattle meat for successful marketing. Discoloration due to the oxidation of myoglobin could negatively impact the utility of cattle meat. The lipid oxidation of meat is considered an important factor which is responsible for the quality deterioration through rancid odor formation and flavor deterioration [72], and in some cases this generates some compounds that can be detrimental to human health as well [73,74]. The rate of oxidation of the lipid depends on the balance between the exogenous and endogenous factors. The presence of oxygen, temperature, and storage condition are the exogenous factors, and fatty acid composition, total lipid content, amount and category of iron presence, reducing compounds (e.g., ascorbic acid), natural antioxidants (e.g., anserine), carnosine and alfa-tocopherol, antioxidant enzymes (e.g., superoxide dismutase), and catalase are the endogenous factors [73,74]. The pH and microbial growth can affect the lipid oxidation or the TBARS value of meat while, in conjunction with them (pH and microbial growth), the presence of energy substrates (glucose or lactate), enzymes, metabolic byproducts in meat, and enzymes generated by microbes can contribute to the lipid oxidation or the spoilage of meat [75,76]. There is a close appositeness between lipid oxidation and myoglobin, and the balance between anti- and pro-oxidants is also related to lipid oxidation in meat. The protection of lipid oxidation occurs due to the antioxidant enzymes, including glutathione peroxidase, superoxide dismutase, and catalase. Susceptibility to lipid oxidation is determined by the content of myoglobin, free ionic iron, and additionally the ferric ion reducing capacity [73,74]. 

Nakyinsige et al. [62] reported a lower TBARS value in the halal slaughter group compared to the stunned group following non-halal slaughtering during the later stage of the storage period, where they explained the lower retention of blood in the carcass in the case of halal slaughter compared with other slaughter types. Higher residual blood in meat elevates the concentration of hemoglobin and heme proteins in meat, where hemoglobin acts on the lipid oxidation phenomena as an eminent promoter [77,78]. Therefore, the reduced retention of blood in the meat of halal slaughter compared with other slaughter methods can result in the reduced oxidation of meat. It can be interpreted as the lower retention of blood in the SSCS group compared to the SSUC group, and the reason for exhibiting a lower TBARS value was due to lower hemoglobin content and the prevention of lipid oxidation. The diminished TBARS value is linked with low free iron content and high ferric ion reducing capacity [73,74]. The difference in heme pigment and the activity of the catalase enzyme shaped the lipid oxidation rate in the case of raw meat [73,74]. It is postulated that the presence of a higher content of heme pigment can generate a higher amount of H_2_O_2_ during the auto-oxidation of oxymyoglobin than that of the presence of a lower amount of heme pigment [73,74], where ferrylmyoglobin is generated by the reaction of H_2_O_2_ and metmyoglobin, and ferrylmyoglobin consequently can cause the commencement of lipid peroxidation [73,74]. Differences in slaughtering followed by captive bolt stunning, with or without physical brain disruption and state of consciousness of animal, might impact the retention of blood in the carcass, which determined the heme and myoglobin content which consequently affected the lipid oxidation in the current study, because in the case of SSUC the animal was in a dead or unconscious state, whereas in the case of SSCS the animal was in a live or conscious state. The aspiration of blood into the lungs and blood lining in the inner space of the trachea, and on the upper space of the bronchi, due to stunning and slaughter variation, could help to explain the variation in blood output in the cases of SSUC and SSCS slaughtering in this study [79,80], which can affect the chemical constituents and color coordination as well as the storage phenomena of muscle.

The pH of muscle can be influenced either in fresh or in storage condition, while the variation in pH value consequently can affect the color of meat. Meat samples with a low pH value had the elevated level of L* and diminished level of a* values compared to that of the high pH value during 7-day post-mortem storage; it was also reported that losses in a* value (red color) along with the period of time were greater to some extent, while there existed a lesser pH value of the muscle samples [70]. There exists an association between muscle pH and muscle redness (muscle color), which happens via the oxidation of oxymyoglobin to metmyoglobin [81]. During the storage condition of meat, the rate of accumulation of metmyoglobin on the surface of beef is concomitant with many intrinsic and extrinsic factors. Intrinsic factors include the muscle type, pH, animal breed, sex, age, diet, and so on, whereas the extrinsic factors include pre- and post-slaughter handling and management [82]. Usually, the oxidation of lipid is initiated by the phospholipid fraction of the cell membrane, which is composed of several unsaturated liposoluble molecules such as cholesterol (prone to lipid oxidation) [83,84]. The differences in the value of TBARS depend on the form of ‘Fe’ catalysts such as myoglobin or free iron [85,86]. Therefore, the variation in liposoluble molecules (cholesterol) and color coordination (myoglobin) might impose on the TBARS value, which seems to be the supporting explanation for the differences between groups. 

### 4.4. Correlation, RSQ, and Intercept among pH, Microbial Loads, and TBARS Value

The pH value can dominate the phenomena and distribution pattern of microorganisms, where microorganisms can influence the oxidation of lipid and protein. Microbes are customarily live in a wider range of pHs and are ascribed as neutrophiles, acidophiles, and alkaliphiles, which usually thrive on the optimal level of pH for further growth and multiplication [87]. The pH may affect the metabolism of microbes and consequently the community structures of microbes by fine-tuning the thermodynamics and kinetics of redox reactions, while the respiration that happens in microbes catalyzes the redox reactions in order to synthesize ATPs. Thus, the respiration rates gamble on the thermodynamic drives, the distinctions between the availability of energy from redox reactions, and the conservation of energy through the respiration process in biological systems [88]. Many redox reactions generate or consume protons; consequently, their free energy yields differ with pH value [89]. If the availability of energy equals or falls below the energy conservation level, the respiration reactions will be thermodynamically unfavorable [90]. In this way, pH may help to sway the breakthrough of microbial respiration and growth, which ultimately will regulate the composition of the certain community. The favorable pH for the growth and development of spoilage-type bacteria in the case of meat or meat products ranges from 5.5 to 7.0, where the formation of slime, structural component degradation, generation of off odors, and changes in appearance were observed in meat as a consequence of microbial growth within the favorable pH range [91]. Canonical correlation analysis advised that the spoilage caused by microbial action, myoglobin autoxidation, protein oxidation, and lipid oxidation unitedly interacted with color and other attributes of meat [92]. Bacterial growth and multiplication are correlated with meat spoilage, where repugnant odors and flavors are generated, discoloration happens, and gas and slime are formed [93]. It was conceded that linear mixed models revealed that microbial spoilage, myoglobin autoxidation, protein oxidation, and lipid oxidation positively impact on yellowness, and they can inversely interact with lightness and redness coloration of meat [92,93]. 

Despite the range of meat pH investigated in the research conducted by Koutsoumanis et al. [94] being relatively narrow (from 5.34 to 6.13), a significant impact of meat pH on the phenomena of growth kinetics of different microbes such as *B. thermosphacta*, *Enterobacteriaceae*, and the *Pseudomonads* category was reported. Similar types of results were reported by Blixt and Borch [95], who proclaimed a significant variation in the growth and multiplication of *Pseudomonads* while the meat pH value exhibited 5.35, relative to that of meat pH at the level of 5.7; other laboratory-based studies stated that *Pseudomonads* growth was unaffected by pH in the range of 5.3 to 7.8. Such types of discrepancy might be imputed to the fact that, in the case of meat, a little variation in pH can be attributed to significant distinction in the concentration of lactate [95] and hence sway the growth of *Pseudomonads*, which are vulnerable to lactic acid [96]. Notably, it was reported by Blixt and Borch [95] that the concentration of lactate was 599 and 946 mg/100 g for meat samples, while the pH value showed 5.7 and 5.35, respectively. Certainly, the modified Arrhenius model for the synergistic aftermath of pH and temperature illustrated better growth of *B. thermosphacta*, *Pseudomonads*, and *Enterobacteriaceae* than that of the single consequences of temperature of the Arrhenius model. Contrary to the bacterial groups, the pH of meat did not influence the growth kinetic phenomena of lactic acid bacteria. In this regard, the phantasm can be explained by the degree of acid tolerance of lactic acid bacteria relative to the other bacterial consortia that cause the spoilage of meat or food items [95,97].

In general, antecedent meat pH value can vary significantly, relying on the feeding and handling of the animal or on other factors influencing the rigor mortis phenomena. The pH data of the investigated meat samples in conjunction with basic particulars on temperature context along with meat storage and shipment were applied to advance the experimental design [98], where it was postulated that, via analogy to the minimum convex polyhedron [99], the polygon that was stated in the model encircles the interpolation region of the suggested model. In opposition to the storage temperature, a negative linear correlation between pH value of meat and ln (μmax × λ) for the *Pseudomonads* category and *B. thermosphacta* category was attended to by Koutsoumanis et al. [94]; it was observed that regression lines were approximately indistinguishable with the predictions for the affiliation of pH value and microbial population and other associated factors. A comparable correlation has also been divulged by Delignette-Muller [100] for other categories of spoilage and pathogenic microorganisms. The interaction between the physiological state and the pH value of meat or food items might be assigned by the physiological stress of the cells influenced by their introduction to a higher degree of an acidic environment. Notably, the elevated concentration of lactic acid in meat with an auxiliary pH may show additional “adaptation work” (i.e., proton pumping by membrane-bound H+-ATPase) required by the cells in order to uplift the midmost pH over an edge value essential for entering into the exponential phase [101]. 

The relativity of the physiological state upon the factors associated with environment other than temperature has been stated by Pin et al. [102], who investigated that in the case of the packaging atmosphere, there exists an ascending correlation between the “adaptation work” of *Yersinia enterocolitica* and CO_2_ concentration. The absence of a correlation between physiological state and the pH value of meat was postulated in the case of lactic acid bacteria and *Enterobacteriaceae*, while in the case of lactic acid bacteria, as quoted in the case of μmax and λ, this result could be blamed to their surpassing degree of acid tolerance relative to the rest of the bacterial groups. A consistent type of findings has been noticed in the case of *Listeria monocytogenes* by McKellar et al. [103], who stated that changes in pH do not prevail the physiological state of the pathogenic microorganisms. The research outputs suggested that the influence of the environmental factors on the physiological state of microbes was attributed not only to the essence of the factor but also to the category of the microbes and their physiology. Thus, from the present study, the correlation of the *Longissimus dorsi* muscle’s pH, microbial load, and TBARS value during 1 d, 1st week, and 2nd week data indicated that the slaughtering following stunning with or without brain disruption and conscious state of KHFC might have an effect. Further detailed biometrics and bioinformatics study can help in discovering such findings. The RSQ values revealed that the data were a good fit for the statistical explanation of *Longissimus dorsi* muscle’s pH, microbial load, and TBARS value during 1 d, the 1st week, and the 2nd week of storage quality following little variation during the slaughtering stage in the case of KHFC in an abattoir. The intercept values revealed that the data were normal for the statistical explanation of *Longissimus dorsi* muscle’s pH, microbial load, and TBARS value during different storage periods based on little variation during slaughtering stages in the case of KHFC in an abattoir, where there was slaughtering after stunning with or without brain disruption and the live and dead conditions of the animal were observed.

## 5. Conclusions

*Longissimus dorsi* muscle’s proximate composition (exempting uplifted total ash content) and cholesterol content did not differ between slaughter types subjected to slaughtering followed by stunning with or without physical brain and spinal cord disruption, and state of consciousness of KHFC (SSCS vs. SSUC). Total fatty acids and their ratios were not affected, irrespective of slaughter types (SSCS vs. SSUC); however, some particular saturated fatty acids, namely lauric, myristic, and myristoleic acid, were diminished in SSCS compared with SSUC. A tendency of diminished microbial population, higher pH value, and downturned TBARS value was exhibited in SSCS compared with SSUC during the 2nd week of storage. The present results suggested that, in comparison to SSUC, the SSCS method assured upgraded storage quality and nutritional aspects to some extent in the case of the *Longissimus dorsi* muscle of KHFC. Thus, SSCS can be applied in the meat industry for the global meat marketing aspects with consumer’s demand and safety concerns.

## Figures and Tables

**Table 1:** Effect of slaughtering with or without state of consciousness on carcass characteristics in Korean Hanwoo Finishing Cattle.

Parameters	SSUC	SSCS	SEM	*p*-Value
Live weight (kg)	721.44	749.27	28.93	0.60
Slaughter weight (kg)	450.50	462.00	14.00	0.61
Carcass yield grade	1.83	2.00	0.36	0.77
Meat quality grade	3.67	3.75	0.49	0.91
Loin eye area (cm^2^)	99.63	103.97	3.37	0.45
Backfat thickness (mm)	17.05	18.16	1.97	0.73
Meat color	4.89	4.96	0.27	0.86
Fat color	3.08	2.87	0.21	0.51
Texture	1.26	1.07	0.08	0.25
Maturity	2.19	2.12	0.07	0.52
ETBW (kg)	43.06	44.89	2.62	0.65

Means were compared based on significance level at *p* < 0.05; SEM: standard error of mean. Marbling score: 1: devoid, 9: very abundant; meat color: 1: bright red, 7: dark red; fat color: 1: creamy white, 7: yellowish; maturity score: 1: young, 3: mature; firmness: 1: firm, 3: soft. Meat color: 1: bright, 7: dark red; fat color: 1: creamy, 7: yellowish; maturity: 1: youth, 2–3: mature. ETBW: estimated blood weight. Korean Hanwoo Finishing Cattle (KHFC) were slaughtered following two methods: (1) SSUC: slaughtering by applying captive bolt stunning, brain and spinal cord disruption, and neck cutting with unconscious state of animal and (2) SSCS: slaughtering by applying captive bolt stunning, without brain and spinal cord disruption and neck cutting with conscious state of animal.

**Table 2:** Effect of slaughtering with or without state of consciousness on *Longissimus dorsi* muscle’s fatty acid pattern in Korean Hanwoo Finishing Cattle.

Parameters	FAN	SSUC	SSCS	SEM	*p*-Value
Fatty acid (g/100 g of total fatty acids)					
Capric acid	C10:0	0.04	0.03	0.001	0.24
Lauric acid	C12:0	0.09 ^a^	0.07 ^b^	0.003	0.001
Myristic acid	C14:0	3.64 ^a^	3.11 ^b^	0.06	<0.0001
Myristoleic acid	C14:1n5	0.31 ^a^	0.24 ^b^	0.01	0.001
Pentadecylic acid	C15:0	0.20	0.24	0.02	0.09
Palmitic acid	C16:0	26.41	24.94	0.58	0.14
Palmitoleic acid	C16:1n7	5.85	5.68	0.07	0.11
Margaric acid	C17:0	0.70	0.38	0.13	0.09
Stearic acid	C18:0	8.40	8.60	0.15	0.40
Oleic acid	C18:1n9	45.75	47.52	0.55	0.05
Linoleic acid	C18:2n6	0.07	0.09	0.01	0.06
Arachidic acid	C20:0	0.07	0.07	0.002	1.00
Alfa-linoleic acid	C18:3n3	0.27	0.28	0.05	0.84
Dihommo-gamma linoleic acid (DGLA)	C20:3n6	0.13	0.16	0.01	0.07
Arachidonic acid	C20:4n6	0.21	0.25	0.02	0.05
Nervonic acid	C24:1n9	0.07	0.07	0.01	0.88
SFA		37.68	39.31	0.72	0.16
UFA		54.22	52.74	0.57	0.12
PUFA		0.75	0.71	0.05	0.52
MUFA		53.46	52.04	0.58	0.13
UFA/SFA		1.44	1.35	0.04	0.15
PUFA/SFA		0.02	0.02	0.001	0.09
MUFA/SFA		1.42	1.34	0.04	0.15

^a, b^ Means in the same row with different superscript letters are significantly different at *p* < 0.05; SEM: standard error of mean. FAN: Fatty acid nomenclature; SFA: Saturated fatty acid; UFA: Unsaturated fatty acid; PUFA: Polyunsaturated fatty acid; MUFA: Monounsaturated fatty acid.

**Table 3:** Effect of slaughtering with or without state of consciousness on *Longissimus dorsi* muscle’s pH in Korean Hanwoo Finishing Cattle during storage at 4 °C.

Parameters	SSUC	SSCS	SEM	*p*-Value
Meat pH				
1 day	5.67	5.68	0.10	0.93
1st week	5.65	5.64	0.04	0.85
2nd week	5.22 ^b^	5.37 ^a^	0.02	0.05

^a, b^ Means in the same row with different superscript letters are significantly different at *p* < 0.05; SEM: standard error of mean.

**Table 4:** Effect of slaughtering with or without state of consciousness on *Longissimus dorsi* muscle’s microbiology in Korean Hanwoo Finishing Cattle during storage at 4 °C.

Parameters	SSUC	SSCS	SEM	*p*-Value
Meat microbiology (log10cfu/g)				
1 day	4.15	4.18	0.57	0.97
1st week	6.97	6.72	0.11	0.20
2nd week	8.01 ^a^	7.67 ^b^	0.10	0.09

^a, b^ Means in the same row with different superscript letters are significantly different at *p* < 0.05; SEM: standard error of mean.

**Table 5:** Correlation between *Longissimus dorsi* muscle’s pH, microbiology, and oxidative stability (TBARS value) in Korean Hanwoo Finishing Cattle.

Parameters	SSUC	SSCS
pH and microbiology		
1 day	0.705	−0.898
1st week	0.488	0.838
2nd week	−1.000	0.953
pH and TBARS		
1 day	−0.857	−0.998
1st week	0.823	0.167
2nd week	1.000	0.985
Microbiology and TBARS		
1 day	−0.970	0.922
1st week	−0.094	−0.398
2nd week	0.990	0.991

**Table 6:** RSQ among *Longissimus dorsi* muscle’s pH, microbiology, and oxidative stability (TBARS value) in Korean Hanwoo Finishing Cattle.

Parameters	SSUC	SSCS
pH and microbiology		
1 day	0.497	0.806
1st week	0.238	0.702
2nd week	1.000	0.908
pH and TBARS		
1 day	0.734	0.996
1st week	0.678	0.028
2nd week	1.000	0.971
Microbiology and TBARS		
1 day	0.941	0.851
1st week	0.009	0.158
2nd week	0.979	0.981

**Table 7:** Intercept among the *Longissimus dorsi* muscle’s pH, microbiology, and oxidative stability (TBARS value) in Korean Hanwoo Finishing Cattle.

Parameters	SSUC	SSCS
pH and microbiology		
1 day	4.807	5.988
1st week	4.426	3.586
2nd week	7.909	1.163
pH and TBARS		
1 day	5.929	5.989
1st week	5.513	5.612
2nd week	4.975	5.170
Microbiology and TBARS		
1 day	5.137	0.710
1st week	7.014	6.924
2nd week	6.525	7.314

## Data Availability

Data is contained within the article.
